# Reliability and validity of the East Asian children's version of mini-MACS in children with cerebral palsy

**DOI:** 10.3389/fresc.2022.997221

**Published:** 2022-11-21

**Authors:** Xiaohui Hou, Huiying Qiu, Liru Liu, Yinhua Li, Lu He, Jinling Li, Hongmei Tang, Kaishou Xu

**Affiliations:** ^1^ Department of Sport and Health, Guangzhou Sport University, Guangzhou, China; ^2^ School of Kinesiology, Shanghai University of Sport, Shanghai, China; ^3^ Department of Rehabilitation, Guangzhou Women and Children’s Medical Center, Guangzhou Medical University, Guangzhou, China

**Keywords:** mini-manual ability classification system, cerebral palsy, reliability, validity, occupational therapy

## Abstract

**Background:**

Mini-Manual Ability Classification System (Mini-MACS) was developed for children with cerebral palsy aged 1–4 years, but its validity and reliability in different cultures are unavailable yet. This study was to determine the reliability and validity of Mini-MACS in East Asian children with cerebral palsy and investigate the correlation between Mini-MACS and Gross Motor Function Classification System.

**Methods:**

One hundred and four East Asian children with cerebral palsy aged 12–48 months were classiﬁed by one of their parents, an occupational therapist, and a physical therapist with Mini-MACS. The results were analyzed for inter-rater reliability by using intraclass correlation coefficient (ICC). The Nine-hole Peg Test was used for the criterion-related validity analysis, and parents retested their children after 2 weeks to evaluate test–retest reliability. Gross Motor Function Classification System levels were also collected to investigate the correlation with Mini-MACS.

**Results:**

Good inter-rater reliability among the occupational therapist, physical therapist, and parents was found [ICC = 0.984 (95% confidence interval, CI, 0.976–0.989), 0.973 (95% CI 0.960–0.982), and 0.966 (95% CI 0.950–0.977), respectively; *p* < 0.01]. The test–retest reliability in parents was almost perfect [ICC = 0.985 (95% CI 0.977–0.990), *p* < 0.01]. Mini-MACS had consistency with the Nine-hole Peg Test (*r* = 0.582, 0.581, and 0.566, respectively; *p* < 0.01). A correlation was found between Gross Motor Function Classification System and Mini-MACS (*r* = 0.626, 0.596, and 0.598, respectively; *p* < 0.01).

**Conclusion:**

The Mini-MACS demonstrates evidence that it is a valid and reliable tool to classify manual ability in East Asian children with cerebral palsy and is also positively related to the Gross Motor Function Classification System.

## Introduction

Cerebral palsy is a common condition that can cause physical disability in children (1). Overall prevalence of children with cerebral palsy was 2.0%–3.5% in the past 40 years, and it might be higher in developing countries (2). About two-thirds of children with cerebral palsy have hand dysfunctions (3), and disorders of body structures of children with cerebral palsy such as secondary musculoskeletal problems may also lead to hand dysfunctions (4). Children with different subtypes of cerebral palsy have various difficulties in manual ability. Furthermore, affected manual ability may be the main reason for limitations in daily activities for children with cerebral palsy, such as eating, drinking, dressing, and grooming (5). For example, children with cerebral palsy may be unable to lift up heavy items with their weak muscle strength or fail to open the door with a key for their limited range of motion. However, children with hand dysfunctions may also affect the development of other functions, including sensory, cognitive, and communication, which possibly damage children's learning tasks and social activities at school (6).

At the age of 1–4 years, children's manual ability rapidly develops, and it may be the appropriate timing for children with cerebral palsy to have interventions and improve their manual ability (7). House classification and bimanual fine motor function classification system have been applied for children with cerebral palsy aged 1–4 years to assess manual ability (8, 9). However, they are not satisfactory because these two assessment tools focus on movements but may ignore the general performance that children show in daily life (10). The Manual Ability Classification System (MACS) was designed for children with cerebral palsy aged 4–18, which is used in 25 countries (11–14). It briefly describes the manual ability of children with cerebral palsy and is a common language for different clinicians. However, the manual ability of children with cerebral palsy has mostly developed during the first 4 years. Thus, the Mini-MACS was developed for children with cerebral palsy aged 1–4 years based on the Manual Ability Classification System (15). Both Mini-MACS and Manual Ability Classification System were developed based on the concept of International Classiﬁcation of Functioning, Disability and Health, Children and Youth Version (ICF-CY) and Gross Motor Function Classification System (GMFCS) (16). The classification systems are useful, as they may distinguish different severities of patients’ function, prognose the development of manual ability of children with cerebral palsy (17), and provide guidance for setting therapeutic goals.

Because of different cultures, customs, and environments, assessment tools usually need to be adapted in different nations and areas so as to be rationally used. However, the reliability of Mini-MACS was only tested in Sweden, and study of reliability and validity was insufficient. So far, the Mini-MACS has been mentioned in few articles. It deserves to be further explored. In addition, correlations among different classification systems such as Gross Motor Function Classification System, Manual Ability Classification System, and Communication Function Classification System were verified in an early study (18) to reveal the interrelation in different functions. A significant correlation between Manual Ability Classification System and Gross Motor Function Classification System indicated their sensitiveness for children with cerebral palsy (18, 19). However, the research by Carnahan et al. indicated that there was no significant correlation between Manual Ability Classification System and Gross Motor Function Classification System, and further research on the correlation has important implications for clinical practice (20). In addition, we found that the correlation study on Mini-MACS and Gross Motor Function Classification System has been unavailable yet. We hypothesized that there was a positive correlation between Mini-MACS and Gross Motor Function Classification System.

Therefore, the objectives of this study were to (1) translate Mini-MACS into Chinese and check the accuracy of the translation; (2) test the reliability and validity of Mini-MACS in East Asian children with cerebral palsy; and (3) evaluate the correlation between Gross Motor Function Classification System and Mini-MACS.

## Methods

### Participants

In this study, one occupational therapist, one physical therapist, children with cerebral palsy, and their parents participated. The research was approved by the Medical Ethics Committee of the Guangzhou Women and Children's Medical Center. This study is registered under registration number ChiCTR-SOR-17014150 All the parents signed informed consents before the test started.

The study recruited 104 children aged 1–4 years, from China and Japan (parents and children were fluent in Chinese), including 80 boys and 24 girls. They were treated with rehabilitation service during March to August 2017. Children in this study were diagnosed with diplegia (*n* = 47), hemiplegia (*n* = 38), quadriplegia (*n* = 10), dyskinesia (*n* = 6), hypotonia (*n* = 2), and ataxia (*n* = 1) (Table 1). Assessors were an occupational therapist and a physical therapist from the Department of Rehabilitation at the Guangzhou Women and Children's Medical Center, and one of the caregivers who took much time with the child in daily life. In this study, all the caregivers were parents.

**Table 1:** Demographic data of the participants (*n* = 104).

Characteristic	
Age in months at recruitment, median (range)	31.9 ± 10.01 (12–48)
Age of 12–23 months, *n* (%)	24 (23.1)
Age of 24–35 months, *n* (%)	39 (37.5)
Age of 36–48 months, *n* (%)	41 (39.4)
Sex, *n* (%)	
Female	24 (23.1)
Male	80 (76.9)
Nationality, *n* (%)	
China	102 (98.1%)
Japan	2 (1.9%)
Gross motor function classification system levels, *n* (%)	
I	45 (43.3)
II	31 (29.8)
III	18 (17.3)
IV	7 (6.7)
V	3 (2.9)
Cerebral palsy types, *n* (%)	
Diplegia	47 (45.2)
Hemiplegia	38 (36.5)
Quadriplegia	10 (9.6)
Dyskinetic	6 (5.8)
Hypotonia	2 (1.9)
Ataxia	1 (1.0)

Children matching the following conditions were recruited: (1) being diagnosed with cerebral palsy; (2) at the age of 1–4 years; (3) being able to understand some easy instructions; (4) having normal or corrected visual and hearing ability (based on the children's hearing and vision screening after birth, as well as reviewing medical history); (5) being accompanied with caregivers. The exclusions included that children (1) suffered from other disease(s) that was(were) not associated with cerebral palsy; (2) had severely fixed contracture or deformity of upper limb(s); and (3) received botulinum toxin injection, baclofen treatment, and orthopedic surgery of upper limb(s) within 6 months. Previous studies have shown that cerebral palsy or “high risk of cerebral palsy” could be accurately predicted before aged 6 months’ corrected age (21). In this study, all the children were diagnosed as cerebral palsy for their obvious motor impairment, abnormal magnetic resonance imaging, or risk factors. Since parents were involved, there were inclusion criteria for them that (1) at least one of the parents was able to communicate and read in Chinese; and (2) parent who participated was the child's caregiver in daily life.

### Instruments

The Mini-MACS has five levels from I to V (15), describing children's different abilities to manipulate objects and their need for assistance or adaptation for daily living activities that were age-appropriate (22). Children at level I were able to handle objects easily and successfully with slight limitation in coordination and precision. Children at level II were able to handle most objects, but with reduced quality and/or speed. Level III means handling objects with difficulties, such as performing slowly and in low quality. At level IV, children handle a limited selection of easily managed objects in simple actions. Children at level V do not handle objects or have very limited ability to perform. At best, they only push, touch, press, or grasp an item and need constant assistance from others. The Mini-MACS especially focuses on the usual performance of children at home, school, and community, instead of the best capacity in relation to manual ability. GMFCS is used to reflect the gross motor function in children with cerebral palsy. Similar to Mini-MACS, it is divided into five levels from I to V, with level I being the best and level V being the worst (23).

The Nine-hole Peg Test was originally introduced as a measure of dexterity by an official publication of the American Society for Occupational Therapy, which was considered as a gold standard measure for manual dexterity and most frequently used for patients in hemiplegia. It was a widely used and valid assessment tool for rehabilitation of neurologic and orthopedic disabilities in adults and children (24–29), and its normative and validation studies in children had been done in 2000 (30). Therapists could classify the children's manual ability not only by asking parents but also through the observation on children's performance in the Nine-hole Peg Test. Therefore, the Nine-hole Peg Test was a suitable tool for the test of criterion-related validity between Mini-MACS and other assessments. The usage and rules of the Nine-hole Test are available on the internet for free (www.physio-pedia.com/Nine-Hole_Peg_Test).

The properties of outcome measurement instruments involved in this study comply with the COnsensus-based Standards for the selection of health Measurement INstruments (COSMIN) (31, 32).

### Procedure

The usage and content of the Mini-MACS was translated into Chinese by the occupational therapist and a pediatric doctor. Then, the physical therapist translated the Chinese version to English before reading the original text. The two therapists compared the back-translation version with the original text and modified the first Chinese version. The occupational therapist and physical therapist did a pretest with five children, using the Chinese version and the original English version. Finally, the four therapists and two pediatric doctors discussed the pretest and modified the translation to make it more appropriate (33). In general, the translation was complied with the original text. Adaptation was made about the grammar for obvious differences between Chinese and English. When parents did not understand the meaning of the content, the occupational therapist would give parents examples based on Asian cultural background, like using chopsticks to eat. The Mini-MACS was made as leaflets finally to help parents understand it quickly. The occupational therapist and physical therapist had a discussion and reached an agreement about each meaning of the five levels and differences between every two adjacent levels before the test. It was to ensure that both the assessors knew how to use the Mini-MACS and did the same explanation for every parent. The therapists and doctors participated in this study all had at least 3 years of professional experience.

During the assessment, the occupational therapist, physical therapist, children, and parents were in the same assessment room, so that the parents and therapists could observe the child at the same time. The entire study was conducted by the same occupational therapist and physical therapist. In addition, we also asked parents to provide videos of their child's manual performance in daily living as an aid to assessment. Children performed the Nine-hole Peg Test before the classification. The occupational therapist guided the children to finish the test. Children practiced once using their unaffected/dominated hand first without time record and then started to be tested separately about their ability of the left and right hand. During the test, the occupational therapist, physical therapist, and parents observed the children's ability of fingers pinch, the range of wrist pronation and rotation, and hand–eye coordination, which provided information about children's manual ability for the classification. The occupational therapist recorded how much time the children spent with each hand.

To learn more about children's manual ability, the occupational therapist asked parents some questions concerning the way in which their children manipulated various objects in daily life, namely, (1) Did the children use their hands with some simple actions, such as drinking, eating, and wearing clothes? (2) Did the children need help from others while handling objects? (3) Did the affected hand(s) limit their most daily activities? (4) How long did the children use both hands probably in one day? (5) How many percentages of time did the child need help from parents when he/she was playing with toys? The five mentioned questions were developed by a group of experienced therapists.

**Figure 1:**
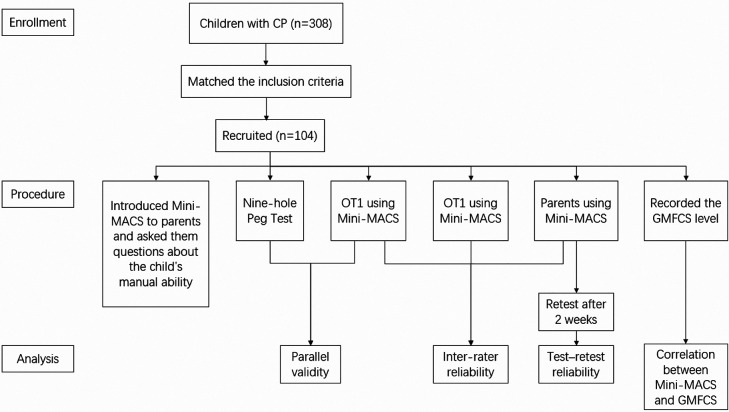
Study flow. CP, cerebral palsy; Mini-MACS, Mini-Manual Ability Classification System; GMFCS, Gross Motor Function Classification System; OT, occupational therapist; PT, physical therapist.

The occupational therapist introduced parents to the Mini-MACS with the leaflet and the physical therapist helped answer parents’ questions about their confusion, including using examples to explain the different levels. After completing the above steps, the occupational therapist, physical therapist, and parents applied the Mini-MACS to evaluate the child, respectively. It meant that each child was assessed for three times by different assessors at the same time. Parents, occupational therapist, and physical therapist recorded the results, respectively. The evaluation results from parents were taken by 84 mothers, 9 fathers, and 11 pairs of parents (who had entered into an agreement). In addition, information of the children was collected, including name, birth date, age, subtypes of cerebral palsy, and Gross Motor Function Classification System levels, which were recorded in their assessment report given by other therapists from Guangzhou Women and Children's Medical Center. Parents were asked to classify their children again after 2 weeks. Many families either did not stay in the hospital for more than 2 weeks or could not come back to the hospital for various reasons. Therefore, the occupational therapist phoned them, read the same leaflet to them, and recorded the results. The leaflet and what to explain were the same as the first assessment. The physical therapist did not participate in this part. The study flow is shown in Figure 1.

### Statistical analysis

We calculated the sample size using the following formula: *N* = *Z*^2^ × *σ*^2^/*d*^2^ [*N*: sample size, *Z* = 0.95, confidence interval (CI); *d* = 0.05, margin of the sampling error; *σ* = 0.5, standard deviation]. We considered the dropout rate of 15%–20%, and the final calculated sample size was 104–108 (34). The data about information of the sample were investigated, including the average age, sex ratio, age ratio, and number of different subtypes of cerebral palsy and different Gross Motor Function Classification System levels.

Reliability of therapists and parents was calculated by SPSS20.0 software (PASW 20, Chicago, IL, United States). As previous studies (14, 15) suggested, the intraclass correlation coefficient (ICC) and the percentage of agreement were to test the therapist–parent agreements. The ICC values ≥0.80 are considered to be acceptable reliability, and *p* value <0.05 is considered statistically signiﬁcant. In addition, a value ≥75% is considered acceptable in the percentage of absolute agreement.

Since the Mini-MACS test results were described as levels I–V and the results of the Nine-hole Peg Test were used time (in seconds), Spearman's correlation analyses were applied to test the criterion-related validity between the two assessment tools. Criterion-related validity was to show the consistency between Mini-MACS and other assessment tools, which had been tested for reliability and validity already. The Mini-MACS was applied to assess usual performance of children's manual ability in daily life, so the best record of the Nine-hole Peg Test obtained by either right or left hand was chosen. In addition, Spearman's correlation analyses were utilized to test the correlation between the Gross Motor Function Classification System and Mini-MACS. Spearman's correlation coefficient is interpreted as follows: *r* ≥ 0.8 very strong relationship; 0.6 ≤ *r* < 0.8 strong relationship; 0.4 ≤ *r* < 0.6 moderate relationship; 0.2 ≤ *r* < 0.4 weak relationship; *r* < 0.2 very weak relationship; and *p* < 0.05 was considered statistically significant.

## Results

### Sample analysis

The average age of 104 children was 31.9 months, ranging from 12 to 48 months. Population in different age stages was evenly distributed. Boys were more than girls in this study, with a percentage of 76.9. In the study, all the Gross Motor Function Classification System levels were included, while levels I–III were more than levels IV–V. As for subtypes of cerebral palsy, most children were in diplegia and hemiplegia. The demographic data of the children are reported in Table 1.

### Reliability among the occupational therapist, physical therapist, and parents

From the comparative analysis between the Mini-MACS results of parents and the occupational therapist, the percentage of agreements was 89.4 and there were 11 disagreements, including 2 disagreements on levels I and II, 6 on levels II and III, 2 on levels III and IV, and 1 on levels IV and V (Table 2A). The ICC values were almost close to 1 (ICC = 0.973, *p* < 0.01). Furthermore, the 95% CI was 0.960–0.982, which meant that data distribution was very concentrated. The above results showed that the classifications of parents and occupational therapist were significantly related.

**Table 2:** Numbers of the Mini-Manual Ability Classification System levels between different assessors.

	Level I	Level II	Level III	Level IV	Level V	Total
**A**. Inter-rater agreements and disagreements (parents and OT)						
OT/parents						
Level I	46	2^a^	—	—	—	48
Level II	—	29	3^a^	-	-	32
Level III	—	3^a^	10	1^a^	—	14
Level IV	—	—	1^a^	6	1^a^	8
Level V	—	—	—	-	2	2
Total	46	34	14	7	3	104
**B**. Inter-rater agreements and disagreements (parents and PT).						
PT/parents						
Level I	43	3^b^	—	—	—	46
Level II	3^b^	30	3^b^	—	—	36
Level III	—	1^b^	10	1^b^	—	12
Level IV	—	—	1^b^	5	1^b^	7
Level V	—	—	—	1^b^	2	3
Total	46	34	14	7	3	104
**C**. Inter-rater agreements and disagreements (OT and PT).						
OT/PT						
Level I	45	3^c^	—	—	—	48
Level II	1^c^	31	—	—	—	32
Level III	—	2^c^	12	—	—	14
Level IV	—	—	—	7	1^c^	8
Level V	—	—	—	—	2	2
Total	46	36	12	7	3	104
**D**. Test–retest agreements and disagreements in parents.						
Parents/retest						
Level I	45	1^d^	—	—	—	46
Level II	1^d^	32	1^d^	—	—	34
Level III	—	2^d^	11	1^d^	—	14
Level IV	—	—	—	7	—	7
Level V	—	—	—	—	3	3
Total	46	35	12	8	3	104

OT, occupational therapist; PT, physical therapist.

^a^ Disagreements between occupational therapist and parents.

^b^ Disagreements between physical therapist and parents.

^c^ Disagreements between occupational therapist and physical therapist.

^d^ Test–retest disagreement.

The diversity of physical therapist and parents’ classifications was totally 14 (86.5% of agreements). There were six disagreements on levels I and II, four on levels II and III, two on levels III and IV, and two on levels IV and V (Table 2B) [ICC = 0.966 (95% CI 0.950–0.977), *p* < 0.01], which also showed a significant correlation.

Reliability between the occupational therapist and the physical therapist was higher than that between therapists and parents. There were only seven disagreements between the occupational therapist and the physical therapist (93.3% of agreements). Four of them were between levels I and II, two were between levels II and III, and one was between levels IV and V (Table 2C) [ICC = 0.984 (95% CI 0.976–0.989), *p* < 0.01].

### Test–retest reliability

As for the reliability of retest, there were seven disagreements (93.3% of agreements). Changes mainly occurred in parents who classified their children as level II at the first test. The result was also almost perfect (Table 2D) [ICC = 0.985 (95% CI 0.977–0.990), *p* < 0.01]. Results of both the initial test and the retest conducted by parents reached a higher degree of consistency.

### Criterion-related validity

Some children could not finish the Nine-hole Peg Test because of severe spasticity or motor impairment. Seventy-seven children completed the Nine-hole Peg Test finally. Consistency was found between children's best record of the Nine-hole Peg Test and the assessment results of Mini-MACS. Spearman's correlation coefficient was 0.582, 0.581, and 0.566, respectively, between the record of the Nine-hole Peg Test and the levels of Mini-MACS taken by the physical therapist, occupational therapist, and parents (*p* < 0.01).

### Correlation between the Gross Motor Function Classification System and the Mini-Manual Ability Classification System

**Figure 2:**
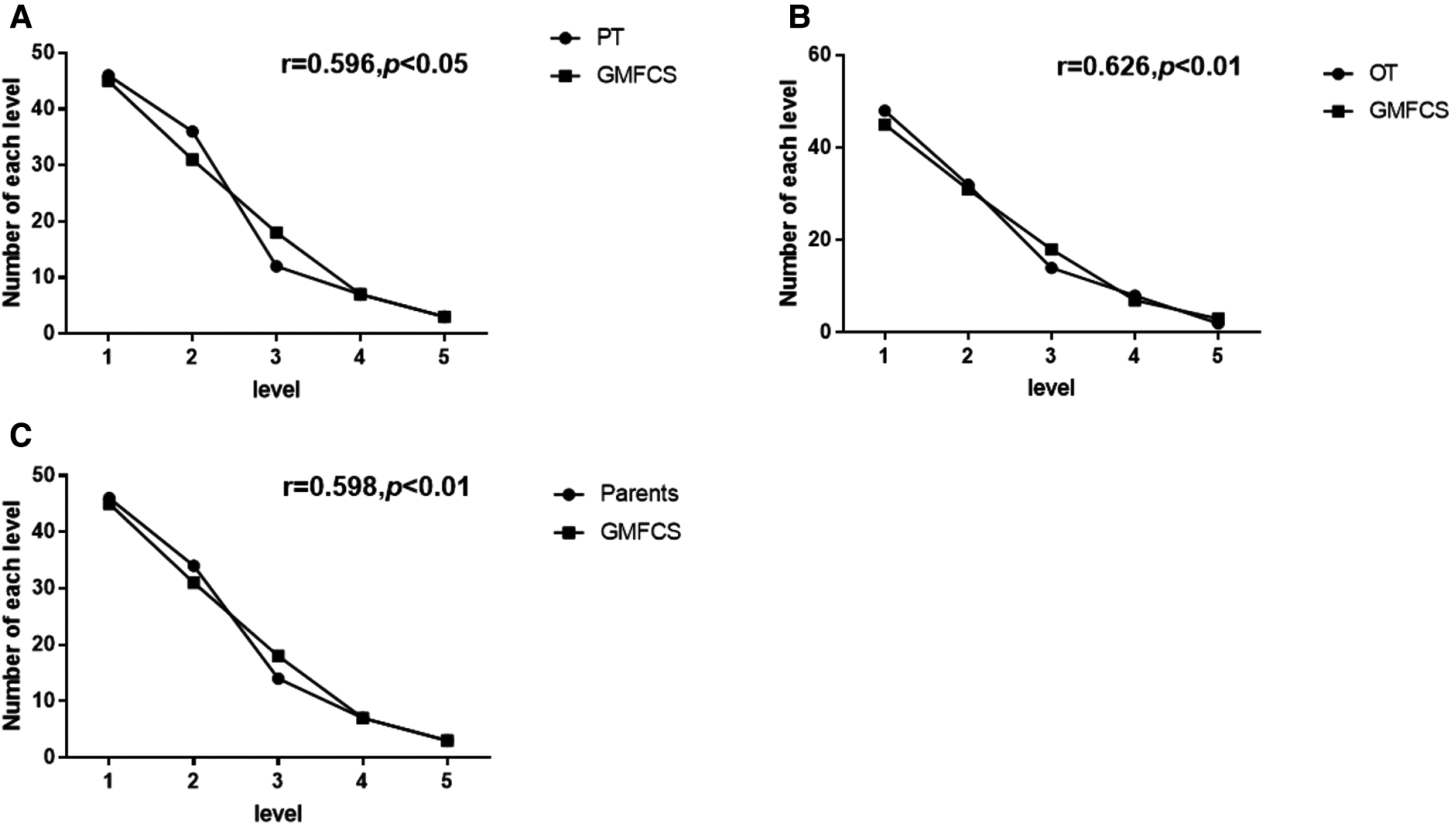
Correlation between the Mini-Manual Ability Classification System (Mini-MACS) and Gross Motor Function Classification System (GMFCS) levels. (**A**) Correlation between GMFCS and Mini-MACS used by the physical therapist (PT). (**B**) Correlation between GMFCS and Mini-MACS used by the occupational therapist (OT). (**C**) Correlation between GMFCS and Mini-MACS used by parents.

In this study, 68.9% of the children in level I of Gross Motor Function Classification System (classified by other therapists, the same below) were in level I of Mini-MACS (classified by occupational therapist, the same below), while 28.9% were in level II. In level II of the Gross Motor Function Classification System, 48.4% children were in level I of Mini-MACS and 35.5% were in level II. Most children in level III of the Gross Motor Function Classification System were in levels II and III of the Mini-MACS. Although children in levels IV and V of the Gross Motor Function Classification System were less, most of these children were classified as levels IV and V of the Mini-MACS (Figure 2). As the data show, there was a correlation between the Gross Motor Function Classification System and the Mini-MACS (classified by the occupational therapist, the physical therapist, and parents; *r* = 0.626, 0.596, and 0.598, respectively; *p* < 0.01; Figure 2).

## Discussion

This study was to assess the reliability of the Mini-MACS between different therapists and between parents and different therapists, to verify the test–retest reliability and criterion-related validity, and to investigate its correlation with the Gross Motor Function Classification System. The results show that Mini-MACS was a reliable and valid assessment tool and could be used in different cultural backgrounds. The Manual Ability Classification System applied for children aged 4–18 years had been assessed for the reliability and validity in the United Kingdom (11), China (35), Turkey (13), Korea (12), and Brazil (14), but the Mini-MACS has not been so familiar to clinicians in the world. As clinicians are increasingly concerned about the early intervention for children with cerebral palsy (36), the development of the Mini-MACS would be very useful and convenient for clinical practice. The study may help clinicians and parents to focus on the manual ability of the children with cerebral palsy at an early stage. Classification systems are beneficial as they have the concrete expressions for different stages of a health condition, instead of descriptions like mild, moderate, and severe (18). Therefore, the Mini-MACS will be a practical tool for clinicians, and will help parents understand their children's condition.

Research on the reliability and validity of the Mini-MACS has not been done in other countries except Sweden. Children from different cultural background have diverse habits and customs, which may influence their development of manual ability. For example, based on the preference of food, children in China and Japan need to use chopsticks to eat, while in some western countries, children tend to use forks and knives to have their meal. In addition, it seems subjective for assessors to classify children through observation and communication with parents. Therefore, reliability and validity studies in different cultural backgrounds are necessary.

In this study, the first step was to make the usage of Mini-MACS more accurate, so the therapist who translated the contents referred from some studies (12, 13, 35) to follow the standard translation process and the usage was discussed and revised by the experts who were professional and experienced in assessments about pediatric rehabilitation. In this study, there was a comprehensive and reasonable sample of 104 children, covering all the subtypes of cerebral palsy and every Gross Motor Function Classification System level. The percentage of different age range was relatively equal (Table 1), which helped underline the sensitivity of the Mini-MACS in younger children, especially those at the age of 1–2 years with immature cognitive ability. To ensure the reliability of classification, the direct caregivers who knew the children well were engaged. The occupational therapist, physical therapist, and parents separately recorded the results at the same time to prevent confusion from each other.

Previous studies that have examined the Manual Ability Classification System for children with cerebral palsy had good consistency and reliability between families and professionals (11). Similarly, Silva et al. demonstrated that fair agreement was observed between the classification performed by the occupational therapist and the classification performed by the parents (14). This study revealed a relatively significant correlation in Mini-MACS results between parents and the therapists in children with cerebral palsy. In the analysis of inter-rater reliability, there were still some disagreements between parents and therapists about the children's Mini-MACS level, which might be related to parents’ education and attitudes/expectations toward their children. There might be two situations: (1) parents who were over optimistic might exaggerate their children's performance; (2) parents who were upset with their children's manual ability might underestimate them. To avoid possible incorrect results, the occupational therapist and physical therapist asked for videos about children's manual performance in daily life or showed other items for children to manipulate like using a crayon to draw on a paper. Fortunately, these situations occurred rarely. In this study, reliability was tested between not only different therapists but also parents and therapists. The Mini-MACS was used to classify children's manual ability in daily life, and parents were most familiar with their children. Therefore, the test conducted by parents was needed.

Disagreements between the occupational therapist and physical therapist were less and occurred when it was difficult to distinguish level II from level III and to match the children's performance and parents’ description. The Nine-hole Peg Test was not difficult for children to figure out how to complete after the demonstration by the occupational therapist. There were previous studies using the Nine-hole Peg Test to test fine motor for children under 4 years old (24, 37). The Nine-hole Peg Test is recommended to evaluate children's fine motor coordination through simple fine motor patterns, including reaching, grasping, carrying, entering, and releasing, which matches the concept of the Mini-MACS. Through the activity, therapists could observe the speed, completion, and how much help the child needed. It could measure hand dexterity in children who had impairment of manual ability (38). The correlation between the Nine-hole Peg Test and Mini-MACS in this study indicates that the Mini-MACS is a valid assessment tool and has consistency with other tools. However, the correlation of these two assessment tools was not high, on the one hand, probably due to the data deficiency of Nine-hole Peg Test, and, on the other hand, because the Nine-hole Peg Test is recommended mainly to assess fine motor coordination, while the MACS considers the general manipulation of objects daily living. As for the test–retest reliability, it was from the perspective of parents, because parents are familiar with their children and they desire to help their children.

Furthermore, early studies had mentioned that there was a high correlation between the Gross Motor Function Classification System and Manual Ability Classification System (*r* = 0.735) (19). Hidecker et al. found a high association between the Gross Motor Function Classification System and Manual Ability Classification System levels in a population of 222 children with hemiplegia and quadriplegia (6). Compagnone et al. also found a significant statistical association between the Manual Ability Classification System and the Gross Motor Function Classification System specifically limited to the lowest level of function (level V) (18). Similar conclusions were partially reached by Park et al. with reference to the relation between the Gross Motor Function Classification System and the Manual Ability Classification System (39). In this study, it is shown that the lower levels of the Gross Motor Function Classification System the children were at, the lower levels of the Mini-MACS they were classified (Figure 2), which suggested that Mini-MACS is also correlated to the Gross Motor Function Classification System. For example, the coordination of upper and lower limbs was needed when a child was climbing, while children having dysfunction in balance could walk better with the help of hands. It was preferable that gross motor and manual ability were interrelated. However, variations of manual ability might be decided by the subtypes of cerebral palsy and age (40). The manual ability of children with diplegia might be better than their gross motor function, so in this study, nearly half of the children in level II of the Gross Motor Function Classification System were classified in level I of the Mini-MACS. The levels of the Gross Motor Function Classification System and Mini-MACS did not correspond one by one, but to some extent they did have correlation from the results of data. Further research on the correlations between the Gross Motor Function Classification System, Mini-MACS, age, and subtypes of cerebral palsy would be necessary.

There was a study to understand the longitudinal development of manual ability in children with cerebral palsy using the Assisting Hand Assessment and Manual Ability Classification System (17). Children in different levels of the Manual Ability Classification System had different rates of development, different stable limits, and reached their stable limits in different ages, which helped predict the development of the manual ability in the future. Thus, the study on the Mini-MACS might be able to have the prediction in an earlier stage.

## Study limitations

The sample was relatively all-round, but it was not well-distributed due to the fact we did not perform a hierarchical sample size calculation. In this study, children in levels IV–V of the Gross Motor Function Classification System were less than those in levels I–III of the Gross Motor Function Classification System and children in hemiplegia and diplegia were more than other subtypes of cerebral palsy. Children in subtypes of cerebral palsy had different dysfunctions, which were related to their manual ability. The small sample size of Japanese children is a limitation of this study, and future studies that include a larger sample of Japanese children living permanently in China are needed to validate the reliability and validity of the Mini-MACS in Asian children. Moreover, it is a limitation that therapists did not conduct assessment for the reliability of test–retest. However, many families could not be back to the hospital for various reasons, so it is not possible to do that. The Nine-hole Peg Test was a convenient and timesaving tool and was easy for children to perform the test. However, it was not a classification system for manual ability, and finding a more suitable tool might be better to test the validity of Mini-MACS. To get relatively accurate data, this study was designed as rigorously as possible. As the Mini-MACS was a relatively subjective assessment, its accuracy might still be influenced by many factors, such as parents’ attitude about the test, children's cooperation, and the translation process.

## Conclusion

The Mini-MACS demonstrates evidence that it is a reliable and valid instrument for classifying the manual skill of children with cerebral palsy aged 1–4 years for the East Asian population and the Mini-MACS is also positively related to the Gross Motor Function Classification System.

## Data Availability

The raw data supporting the conclusions of this article will be made available by the authors, without undue reservation.
